# Dual anti-CTLA-4 and anti-PD-1 blockade in metastatic basal cell carcinoma

**DOI:** 10.1038/s41698-024-00798-1

**Published:** 2025-01-24

**Authors:** Sandip P. Patel, Eleanor Cano-Linson, Young Kwang Chae, Shiruyeh Schokrpur, Christopher D. Lao, Benjamin C. Powers, Adrienne I. Victor, Adedayo A. Onitilo, Sarah Shin, Naoko Takebe, Sara Threlkel, Christine M. McLeod, Helen X. Chen, Elad Sharon, Megan Othus, Christopher W. Ryan, Charles D. Blanke, Razelle Kurzrock

**Affiliations:** 1 University of California at San Diego Moores Cancer Center, La Jolla, CA USA; 2 SWOG Statistical Center, Seattle, WA USA; 3 Fred Hutchinson Cancer Research Center, Seattle, WA USA; 4 Northwestern University, Chicago, IL USA; 5 University of California at Davis, Sacramento, CA USA; 6 University of Michigan, Ann Arbor, MI USA; 7 University of Kansas Cancer Center, Overland Park, KS USA; 8 University of Rochester, Rochester, NY USA; 9 Marshfield Medical Center—Weston, Weston, WI USA; 10 National Cancer Institute, Developmental Therapeutics Clinic, Bethesda, MD USA; 11 SWOG Data Operations Center/ Cancer Research And Biostatistics, Seattle, WA USA; 12 National Cancer Institute, Investigational Drug Branch, Cancer Therapy Evaluation Program, Bethesda, MD USA; 13 Dana-Farber/Harvard Cancer Center, Boston, MA USA; 14 Oregon Health & Science University, Portland, OR USA; 15 SWOG Group Chair’s Office, Oregon Health & Science University, Knight Cancer Institute, Portland, OR USA; 16 Present Address: Bristol Myers Squibb, New York, NY USA

**Keywords:** Basal cell carcinoma, Outcomes research

## Abstract

We report the basal cell cancer (BCC) cohort of the SWOG/NCI 1609 *Dual* Anti-CTLA-4 & Anti-PD-1 blockade in *Rare Tumors* (DART), a phase II prospective, multicenter basket trial of nivolumab and ipilimumab. The primary endpoint was objective response rate (ORR) (RECIST v1.1). Overall survival (OS), progression-free survival (PFS), and toxicity were secondary endpoints. Sixteen patients with advanced/metastatic BCC were evaluable. The ORR was 31% (95% CI, 19–50%), and the 12-month OS, 75% (95% CI, 57–100%). Median PFS was 9.3 months (95% CI, 3.3–NA). Of 15 patients evaluable for clinical benefit, five partial responses (PRs) and five stable disease >6 months (total = 10/15 (66.7%)) were seen. The most common toxicities included fatigue (37.5%), pruritis (31.3%), and diarrhea (25%). In patients with advanced/metastatic BCC, ipilimumab and nivolumab produced an ORR of 31% and prolonged (>6 months) PFS in 73% of patients, with seven PFS/iPFS of >1 year, including one with prior anti-PD-1. ClinicalTrials.gov ID: NCT02834013 (Registered 7/15/2016; https://clinicaltrials.gov/ct2/show/NCT02834013).

## Introduction

Dual immune checkpoint blockade targeting PD-1 and CTLA-4 has revolutionized the management of metastatic melanoma and proved effective in other cancers, such as non-small cell lung cancer and renal cell carcinoma^1–3^. However, the benefit of these therapy combinations in rare tumors has been largely difficult to ascertain, secondary to a lack of clinical trials for these cancers. The SWOG 1609 DART, a basket immunotherapy trial focused on assessing the utility of these agents for rare tumors using a combination of ipilimumab and nivolumab was developed for this purpose. This trial consists of 53 cohorts across more than 1000 sites in the United States at its peak, highlighting the unique ability of cooperative groups and large basket trials to investigate rare and ultra-rare tumors. Prior studies have shown the power of this approach, such as demonstrating a 26–44% ORR in patients with high-grade non-pancreatic neuroendocrine neoplasms (NEN)^4,5^. The current study addresses the results for patients with locally advanced or metastatic BCC. Combination immunotherapy approaches targeting PD-1 and CTLA-4 using ipilimumab and nivolumab have led to their use in a variety of tumors types, including melanoma, non-small cell lung cancer, renal cell carcinoma, esophageal squamous cell carcinoma, microsatellite-instability-high or mismatch repair deficient colorectal cancer, malignant pleural mesothelioma, and hepatocellular carcinoma^1–3,6–9^. In this basket study, we examine the potential benefit of this treatment combination applied to a variety of rare tumors. We elected to utilize the lowered dosing of ipilimumab 1 mg/kg every 6 weeks to attempt to optimize the balance of toxicity and efficacy. This dose has been effective in larger studies, such as CheckMate-227^2^, as well as shown notable responses in other groups of the SWOG1609^4,5,10–13^. Of note, the results from other arms of this trial have established standard-of-care approaches, such as in the context of high-grade nonpancreatic neuroendocrine tumors^4,5^.

BCC is diagnosed in an estimated 3.6 million patients in the United States annually, making it the most common form of skin cancer according to The Skin Foundation. However, only rarely do patients develop locally advanced, unresectable, or metastatic disease, requiring systemic treatment^14–16^. Inhibitors of the hedgehog pathway, such as vismodegib and sonidegib have demonstrated impressive response rates approaching approximately 60%, with some patients achieving complete responses^17–19^. However, these agents can be poorly tolerated, with progressive toxicity based on the extent of exposure. Patients achieving complete response initially often do not show extended durable benefit, with nearly half relapsing, requiring further therapy^20^. There remains a critical need to establish additional effective alternative therapy strategies for patients intolerant or unable to be treated by these agents. Cemiplimab (an anti-PD-1 agent) has also been approved for treatment of advanced or metastatic BCC, but responses to this single-agent immune checkpoint blockade (ICB) have been modest, with 6% complete and 25% partial responses in the hedgehog inhibitor-failed setting^21^.

The current DART cohort assesses the outcome of dual low-dose anti-CTLA-4 therapy and anti-PD-1 therapy, specifically utilizing the combination of ipilimumab and nivolumab, in the management of patients with metastatic basal cell carcinoma. These approaches seek to exploit the role of these two immune checkpoints at different stages of the immune activation cascade, with disruption of CTLA-4 at the sites of T-cell priming and PD-1 during the function of activated T-cells^22,23^. This synergistic immune activation can potentially increase primary responses or unlock responses in patients with acquired resistance to anti-PD-1 treatment.

## Results

### Patient characteristics

Sixteen eligible patients from National Clinical Trial Network (NCTN) institutions were registered between January of 2018 and September of 2020 (Table 1). Their median age was 67 years (range, 52–80 years), with a male preponderance (81%). Performance status trended toward 0 (62%) with the remainder of patients having a performance status of 1. The most common sites of primary disease included shoulder (3 patients), followed by lip, neck, and scalp (2 patients each, respectively). All but one patient had metastatic disease. Fourteen of the patients self-reported prior treatment with sonic hedgehog inhibitors. Six patients reported treatment with additional systemic agents, including but not limited to carboplatin, paclitaxel, and pembrolizumab. The median line of prior therapy was one (range 0–5).

**Table 1:** Characteristics of patients with metastatic basal cell cancers

Median age (range)	67 years (52–80 years)
Gender:	
Female Male	3 (19%) 13 (81%)
Performance Status:	
0 1	10 (62%) 6 (30%)
Ethnicity:	
Hispanic Not Hispanic	0 (0%) 16 (1--%)
Race:	
White Unknown race	15 (94%) 1 (6%)
Median number of prior therapies (range)	1 (0-6)
Number of patients who had prior immunotherapy Number of patients who had prior Sonic Hedgehog inhibitors	1 (6%) 14 (88%)
Primary site:	
Chest wall Left foot Lip Neck Prostate Scalp Scrotal Shoulder Temple Vulva Skin (location not clear)	1 (6%) 1 (6%) 2 (12%) 2 (12%) 1 (6%) 2 (12%) 1 (6%) 3 (19%) 1 (6%) 1 (6%) 1 (6%)
Benefit and response summary^**a**^	
Confirmed partial response Immune partial response Clinic benefit (stable disease for >6 months)	5 (31%) 1 (6%) 5 (31%)

^a^One patient was not evaluated for stable disease > 6 months because she had ongoing stable disease at 97+ days.

### Toxicities

Twelve of the patients (75.0%) experienced any grade adverse events and four (25.0%) experienced grade 3-4 adverse events at least possibly related to drug (Table 2). Two patients (12.5%) experienced adverse events leading to discontinuation of therapy. The most common any-grade adverse event was fatigue (37.5%), followed by pruritis (31.3%), and diarrhea (25.0%). The three grade 3-4 adverse events were diarrhea (12.5%), increased lipase (6.3%), and increased serum amylase (6.3%). All grade 3-4 adverse events were immune-mediated toxicities. There were no episodes of immune-mediated pneumonitis or myocarditis. There were no grade 5 adverse events in this cohort.

**Table 2:** Treatment-related adverse events (*n* = 16 patients)

	Any grade	Grade 3–4	Grade 5
Any	12 (75.0%)	4 (25.0%)	0 (0.0%)
Serious	1 (6.3%)	1 (6.3%)	0 (0.0%)
Led to Discontinuation	2 (12.5%)	2 (12.5%)	0 (0.0%)
Lead to Death	0 (0.0%)		0 (0.0%)
>10% of Patients			
Fatigue	6 (37.5%)	0 (0%)	0 (0.0%)
Pruritus	5 (31.3%)	0 (0%)	0 (0.0%)
Diarrhea	4 (25.0%)	2 (12.5%)	0 (0.0%)
Anemia	3 (18.8%)	0 (0%)	0 (0.0%)
Aspartate aminotransferase increased	3 (18.8%)	0 (0%)	0 (0.0%)
Rash maculo-papular	3 (18.8%)	0 (0%)	0 (0.0%)
Lipase increased	2 (12.5%)	1 (6.3%)	0 (0.0%)
Serum amylase increased	2 (12.5%)	1 (6.3%)	0 (0.0%)
Alanine aminotransferase increased	2 (12.5%)	0 (0%)	0 (0.0%)
Alkaline phosphatase increased	2 (12.5%)	0 (0%)	0 (0.0%)
Anorexia	2 (12.5%)	0 (0%)	0 (0.0%)
Arthralgia	2 (12.5%)	0 (0%)	0 (0.0%)
Colitis	2 (12.5%)	0 (0%)	0 (0.0%)
Creatinine increased	2 (12.5%)	0 (0%)	0 (0.0%)
Dehydration	2 (12.5%)	0 (0%)	0 (0.0%)
Dry mouth	2 (12.5%)	0 (0%)	0 (0.0%)
Generalized muscle weakness	2 (12.5%)	0 (0%)	0 (0.0%)
Hypothyroidism	2 (12.5%)	0 (0%)	0 (0.0%)
Rash acneiform	2 (12.5%)	0 (0%)	0 (0.0%)
Immune-mediated	11 (68.8%)	3 (18.8%)	0 (0.0%)
Pruritus	5 (31.3%)	0 (0%)	0 (0.0%)
Diarrhea	4 (25.0%)	2 (12.5%)	0 (0.0%)
Aspartate aminotransferase increased	3 (18.8%)	0 (0%)	0 (0.0%)
Rash maculo-papular	3 (18.8%)	0 (0%)	0 (0.0%)
Lipase increased	2 (12.5%)	1 (6.3%)	0 (0.0%)
Serum amylase increased	2 (12.5%)	1 (6.3%)	0 (0.0%)
Alanine aminotransferase increased	2 (12.5%)	0 (0%)	0 (0.0%)
Arthralgia	2 (12.5%)	0 (0%)	0 (0.0%)
Colitis	2 (12.5%)	0 (0%)	0 (0.0%)
Hypothyroidism	2 (12.5%)	0 (0%)	0 (0.0%)
Hyperthyroidism	1 (6.3%)	0 (0%)	0 (0.0%)

### Outcomes

**Fig. 1:**
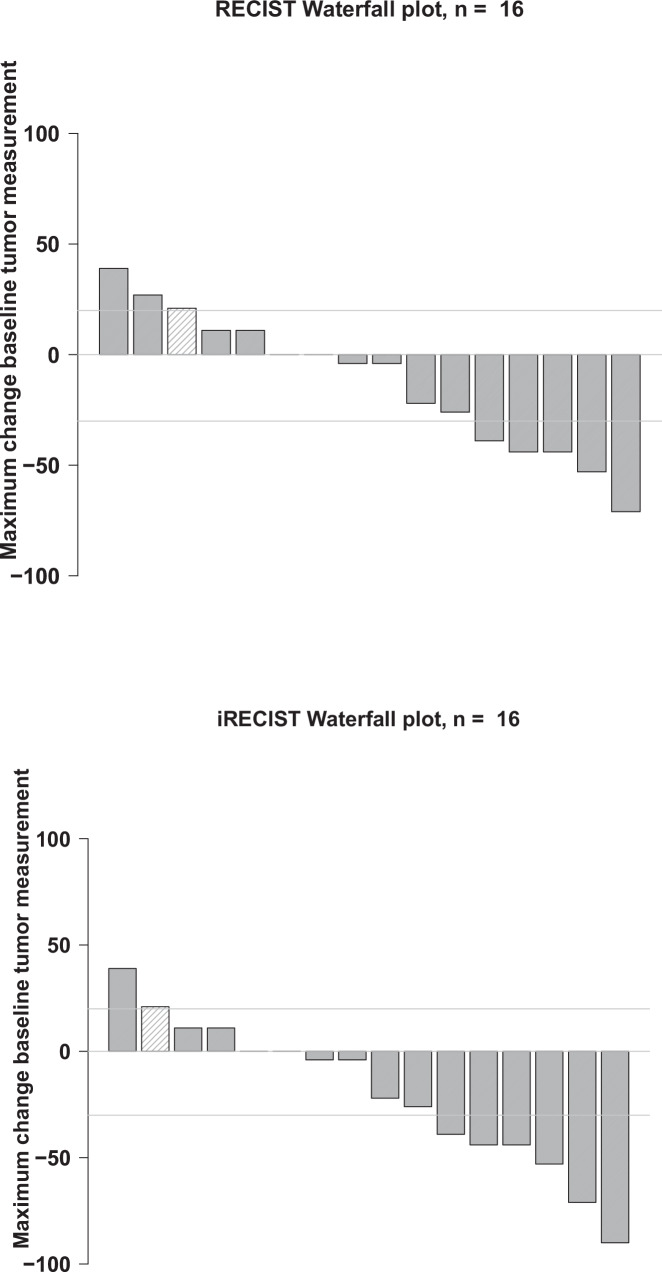
Top Panel: Waterfall plot of tumor measurements. Horizontal grey lines indicate criteria for progression (top) and response (bottom) by RECIST v1.1. Each column represents the maximum change in tumor measurement for one patient. Bottom Panel: iRECIST Waterfall plot. Horizontal grey lines indicate criteria for progression (top) and response (bottom) by iRECIST. Each column represents the maximum change in tumor measurement for one patient.

**Fig. 2:**
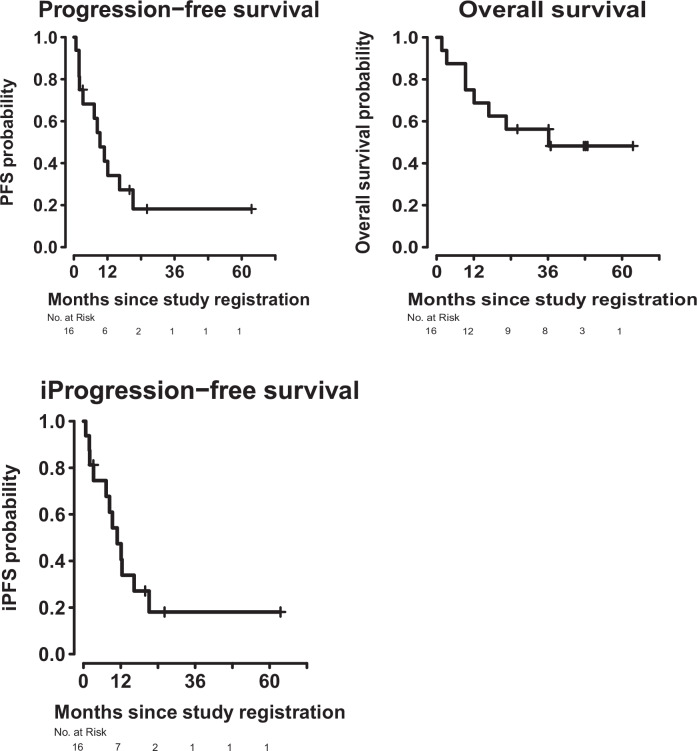
Top Panel: RECIST v1.1 Progression-free and overall survival. Kaplan-Meier curves for RECIST v1.1 PFS (left) and OS (right). Bottom Panel: iRECIST Progression-free survival. Kaplan-Meier curve for iRECIST v1.1 PFS.

**Fig. 3:**
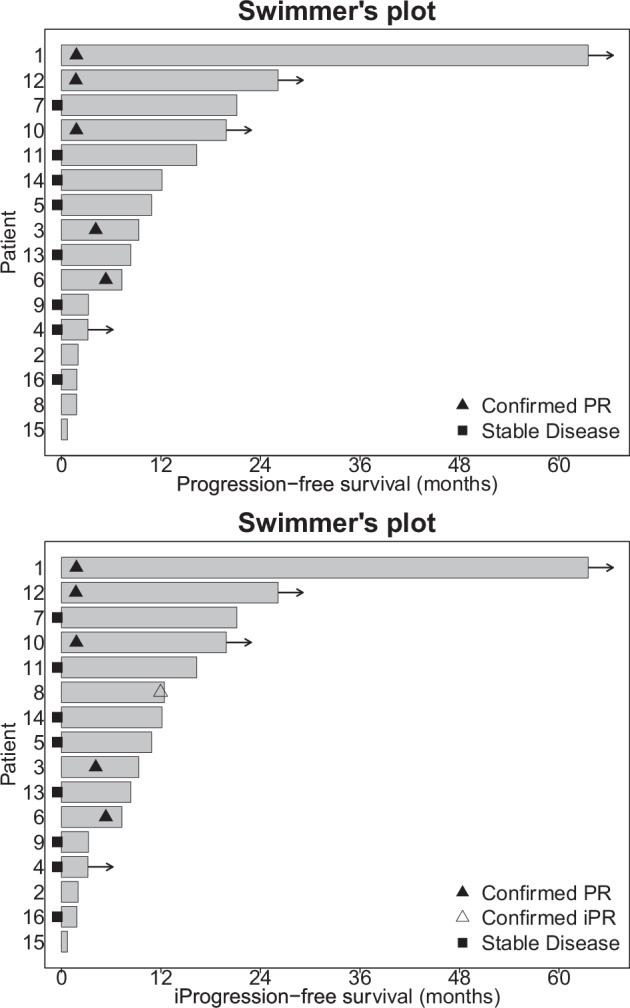
Top Panel: RECIST v1.1 Swimmer’s plot. Each row represents the duration of PFS for a single patient. Circle, triangle, and square symbols denote confirmed PR, unconfirmed PR, and stable disease, respectively. Bottom Panel: iRECIST Swimmer’s plot. Each row represents the duration of PFS for a single patient. Circle, triangle, crossed circle, and square symbols denote confirmed PR, unconfirmed PR, confirmed iPR, and stable disease, respectively.

Five of the 16 patients attained confirmed partial responses (PRs) (31%). Of 15 patients evaluable for clinical benefit (includes benefit (stable disease) of over 6 months) (one patient had ongoing stable disease at 97+ days and was not considered evaluable for this parameter), five achieved PRs and five stable disease (SD) for over six months (total = 10/15 (66.7%)) (Table 1 and Fig. 1). PFS in these 10 patients was 7.4, 8.5, 9.5, 11, 12.3, 16.6, 20.2+, 21.5, 26.5+, and 66.4+ months. An additional patient demonstrated an iPFS of 12.6 months. Kaplan-Meier survival curves are presented for this cohort in Fig. 2. The median OS was 36.2 months (95% CI 12.1–NA) and median PFS was 9.3 months (95% CI 3.3-NA). Six-month OS was 88% (95% CI 73–100) and 12-month OS was 75% (95% CI 57–100). Six-month PFS was 68% (95% CI 49–96). A swimmer’s plot representation of the RECIST responses is presented in Fig. 3. Patients were also assessed via iRECIST. Median iRECIST PFS was 10.9 months (95% CI 7.3–NA) and can be seen on Fig. 2, bottom panel. Six-month iPFS was 74% (95% CI 56–100). A waterfall plot summarizing the iRECIST findings can be found in Fig. 1, bottom panel. One patient who showed progression via RECIST demonstrated confirmed iPR, as seen in Fig. 3, bottom panel. One patient (case #8, Supplementary Table 1) who had had a prior anti-PD-1 agent achieved an iPR with an iPFS of 378 days on the combination of ipilimumab and nivolumab. This patient demonstrated a radiologic response on pembrolizumab but had subsequent mixed results on future scans. His pembrolizumab was originally held due to extensive bug bites but not re-started secondary to progression in some lesions on these mixed scans.

## Discussion

Sonic hedgehog inhibitors form one of the foundations of treatment for locally advanced or metastatic BCCs. However, despite response rates ranging from 8% to 56%, most patients eventually progress or develop prolonged, difficult-to-tolerate toxicities^17–19^. Additionally, responses in patients with metastatic, as opposed to locally advanced, disease have been inferior and are maintained only through continued oral treatment. Additional treatment approaches are critically needed for patients whose disease shows primary or secondary resistance to sonic hedgehog inhibitors and those who are intolerant to this class of medications. Cemiplimab has been approved for the treatment of locally advanced or metastatic basal cell carcinoma, but with less than a third of patients showing responses, there remains a need for more effective treatment options.

The efficacy of single-agent immunotherapy targeting PD-1 has been explored in several recent studies. Stratigos et al. explored the efficacy of single-agent cemiplimab (anti-PD-1) in a phase II study in patients with locally advanced or metastatic BCC following treatment with or intolerant to a sonic hedgehog inhibitors agent^21^. Similar to our findings, they saw a 31% ORR (95% CI 21–42%) in a larger group of 84 patients. Unexpectedly, the rate of grade 3-4 adverse events reported was markedly higher than this study, nearly double at 48%. In a smaller, but similar study, single-agent pembrolizumab, alone or in combination with vismodegib, was assessed for efficacy and tolerability in patients with advanced BCC^24^. Nine patients received pembrolizumab monotherapy, with an ORR of 44% (95% CI 15–65%). Notably, the dual therapy group showed a lower ORR, though this finding was not statistically significant. They reported three grade 3 adverse events amongst the 16 patients in this study. Another phase II study investigated the safety and efficacy of nivolumab as a single agent in advanced BCC, following progression on or intolerance to sonic hedgehog inhibitors^25^. This study observed a 12.5% rate of CR and 18.8% rate of PR, combining for an ORR of 31.3%. In this study population, approximately 40% of adverse events were grade 3-4, with any adverse event affecting 28% of the patients.

The primary endpoint of ORR of 31.5% and notable secondary endpoints of PFS, OS, and safety reported in this study are consistent with those seen in the above single-agent studies. It is therefore not clear that the addition of ipilimumab to nivolumab enhances these outcomes. However, the current study also showed that of 15 patients evaluable for clinical benefit (includes stable disease over six months and iPR), 11 showed salutary effects (73.3%) with seven of the patients showing benefit for more than one year and one patient ongoing at over five years. Notably, the safety profile in our study was comparable to the above studies, indicating that the addition of lowered dose CTLA-4 targeted treatment did not result in significantly higher toxicity here. Also, of interest, one patient who had had a prior anti-PD-1 agent and demonstrated radiologic response followed by progression on scans achieved an iPR with an iPFS of 378 days on the combination, suggesting the possibility that dual immunotherapy with an anti-PD-1 and anti-CTLA-4 can recapture responses after failure of single anti-PD-1 agent. Of note, this patient was on pembrolizumab for seven months with radiologic response prior to showing progression, indicating the development of secondary resistance, which was overcome through our treatment. As there is a great need for additional treatment options for metastatic basal cell carcinoma patients following progression on sonic hedgehog inhibitors and single-agent PD-1-targeted therapy, this combination treatment warrants further evaluation as salvage therapy in this population.

Weaknesses of this study include it being a single-arm study, unblinded, and with a small sample size. Additionally, responses were assessed by local, as opposed to central, radiologic review for the primary outcome, ORR by RECIST v1.1. With the small numbers, this study was not sufficiently powered to assess a survival advantage of this approach.

Overall, this study supports the consideration of ICB in the treatment of advanced BCC. Further work is needed to ascertain the role of ipilimumab plus an anti-PD-1 agent versus an anti-PD-1 agent alone. There is also a need for further studies to evaluate additional therapies that may be combined with PD-1 targeted ICB, as well as biomarkers for patient selection, to improve the response rates and durations for patients with this rare condition.

## Methods

This trial was conducted through the National Cancer Institute (NCI)-supported SWOG Cancer Research Network’s Early Therapeutics and Rare Cancers Committee. It is a multicenter, open-label, basket study for the assessment of combination nivolumab and ipilimumab in rare cancers. Agents were provided through the Cancer Therapy Evaluation Program (CTEP) of the NCI Cooperative Research and Development Agreement with Bristol-Myers Squibb. All protocols and amendments were reviewed and approved by SWOG, the NCI, and the NCI Central Institutional Review Board. All research was performed consistent with the Declaration of Helsinki. Informed consent to participate in the study was obtained from all participants.

### Rationale for population

Rare cancers for this basket study were defined as an incidence of less than 6 in 100,000 annually. Fitting this condition, the cohort of locally advanced or metastatic BCC was opened as part of this study. Local pathology reports were collected at individual sites and reviewed centrally by the SWOG team.

### Patient selection

Enrolled patients were at least 18 years of age, had a hemoglobin ≥8 g/dL, platelets ≥75,000/mcL, absolute neutrophil count ≥ 1000/mcL, creatinine clearance ≥ 50 mL/min, total bilirubin ≤2.0 x institutional upper limit of normal (IULN), TSH or free T4 serum ≤ IULN, AST and ALT ≤ 3.0 x IULN, and adrenocorticotropic hormone (ACTH) ≤ IULN. Women of childbearing age were required to have a negative serum pregnancy prior to participation. They were also expected to practice adequate birth control during the duration of the trial.

### Treatment and monitoring

Treatments included nivolumab 240 mg intravenously (IV) every two weeks and ipilimumab 1 mg/kg IV every six weeks. Patients continued treatment until disease progression, treatment delay for any reason >56 days, unacceptable or immune-related toxicity with inability to tolerate prednisone levels less than 10 mg daily, symptomatic deterioration, or at the patient’s request.

Assessments of patients via history and physical, with attention to developing toxicities were performed at 6-week intervals at the start of each cycle. Laboratory assessment included complete blood count, comprehensive metabolic panel, thyroid stimulating hormone, free thyroxine, adrenocorticotropic hormone, lipase, and cortisol. Computed tomography scans to assess disease status were performed prior to initial treatment, then subsequently at weeks eight, 16, 24, and then every 12 weeks until patients progressed.

### Statistical methods and outcomes

The primary endpoint was assessment of the ORR, a combination of the confirmed complete response and partial response rates [CR and PR, respectively]) based on RECIST v1.1 criteria by local assessment. We utilized a two-stage design to evaluate our null hypothesis of an ORR ≤ 5% against ≥ 30%, an alternative hypothesis representing a potentially clinically meaningful difference in refractory solid tumors. The first stage included six patients. If one or more patients in the initial stage experienced a PR or CR, then an additional ten patients were enrolled in the study, for a total of 16 patients. Two or more patients experience a confirmed response would reject the null hypothesis (one-sided alpha = 13%, power = 87%). SWOG studies typically enroll an additional 5–10% to account for potentially ineligible patients. The secondary objectives were to estimate PFS, OS, ORR by immune-related RECIST (iRECIST), PFS by iRECIST, and toxicity assessment by CTCAE v5.0. Follow-up data are as of January 19, 2024.

PFS was measured from start of therapy on protocol to the first of date of progression according to RECIST v1.1 or death from any cause, with patients last known to be alive without progression censored at the date of last contact. OS was measured from initial trial enrollment to date of death from any causes, with patients known to be alive censored at the date of last contact. Estimates of PFS and OS were calculated by Kaplan-Meier method. Confidence intervals for the medians were generated by the Brookmeyer and Crowley method^26^, and those for point estimates (such as 6-month OS) were calculated with the log-log transformation.

## Supplementary information

Supplementary Table 1

## Data Availability

All datasets and R code to reproduce analyses are available by request following SWOG procedures (Policy 43: Requests for Participant Data): https://www.swog.org/sites/default/files/docs/2019-12/Policy43_0.pdf.
