# Supplementary material for: Dual anti-CTLA-4 and anti-PD-1 blockade in metastatic basal cell carcinoma
Source: NPJ Precis Oncol. 2025 Jan 24;9:24. doi: 10.1038/s41698-024-00798-1 (PMC11759674; doi:10.1038/s41698-024-00798-1)
Supplement: Supplementary file 1 — Supplementary Table 1 [file 41698_2024_798_MOESM1_ESM.pdf]

Supplementary Table 1: Detailed patient characteristics and RECIST responses

Patient Number	Age (years)	Gender	Best response	Days to best response	% change in tumor size	Progression	Death	Progression-free survival
1	64	M	Confirmed PR	111	-39	No	No	1933+
2	76	M	Progression	-	+39	Progressed	Died	61
3	79	F	Confirmed PR	174	-53	No	Died	284
4	79	F	Stable Disease	-	-4	No	No	97+
5	52	M	Stable disease	-	-22	Progressed	No	331
6	75	F	Confirmed PR	163	-44	Progressed	Died	222
7	56	M	Stable disease	-	-26	Progressed	No	644
8*	54	M	Progression (best response by iRECIST was iPR)	-	-	Progressed	Died	56 days (iPFS=378 days)
9	80	M	Stable Disease	-	0	No	Died	99
10	72	M	Confirmed PR	115	-44	Progressed	No	605+
11	74	M	Stable Disease	-	0	Progressed	No	497
12	59	M	Confirmed PR	109	-71	No	No	795+
13	69	M	Stable Disease	-	-4	Progressed	No	255
14	52	M	Stable Disease	-	+11	No	Died	369
15	65	M	Symptomatic deterioration	-	-	Progressed	Died	22
16	64	M	Stable Disease	-	+11	Progressed	Died	57

*This patient (case #8) had prior anti-PD-1 monotherapy
